# Select them well, train them better! Psychological combat readiness in the German army special forces

**DOI:** 10.3389/fpsyt.2026.1774426

**Published:** 2026-03-25

**Authors:** Pablo Mair, Moritz Michels, Daniela da Silva, Karl-Heinz Renner, Ulrich Wesemann

**Affiliations:** 1 Charité – Universitätsmedizin Berlin, Berlin, Germany; 2 German Army Special Forces Command, Calw, Germany; 3 Bundeswehr Joint Force Command, Berlin, Germany; 4 Munich University of the Federal Armed Forces, Neubiberg, Germany; 5 Department for Psychiatrie und Psychotherapie, Military Hospital Berlin, Berlin, Germany

**Keywords:** military personnel, functional work attitude, hardiness, human performance optimization, performance psychology, psychological combat readiness, special forces, unit cohesion

## Abstract

**Introduction:**

Psychological Combat Readiness (PCR) refers to a mindset designed to enhance the mental performance and operational preparedness of military and law enforcement special forces. The concept comprises five core components: *Psychological Stability, Hardiness*, *Functional Work Attitude*, *Comradeship and Team Orientation (Cohesion)* and *VUCA competence*—characterized by volatility, uncertainty, complexity, and ambiguity. The first part of the present study aimed to investigate the trainability of PCR during the basic Special Operation Forces (SOF) training phase of the German Army Special Forces Command (KSK). In particular, it examined whether PCR predicts tactical and military performance parameters such as Close Quarter Battle and hand-to-hand combat training. The second part of the study explored whether PCR could predict commando eligibility in the KSK.

**Method:**

Candidates (*N* = classified) of the German Army Special Forces Command (KSK) were followed longitudinally from 2023 to 2025 during their basic SOF training. Psychological Combat Readiness was assessed with the self-reported 50-items PCR-scale at three assessment periods: at the beginning of the basic SOF training, after 15 months, and after 24 months. Participants in the training cohort were divided into two groups: an experimental group, which received PCR training, including modules on *Hardiness*, *Comradeship and Team Orientation*, and *Performance Optimization;* and a control group, which was trained in progressive muscle relaxation and breathing techniques. After 15 and 24 months, a rMANOVA was conducted to examine changes in the PCR factors and to compare group performance in Close Quarter Battle (CQB) and hand-to-hand combat training. Finally, the initial PCR factors were analyzed using binary logistic regression to determine their predictive value for successful completion of the commando qualification after two years.

**Results:**

The training of PCR showed significant changes in *Hardiness* and *Functional Work Attitude* for the experimental group after 15 and 24 months. In addition, the training compared to the control group showed significantly higher scores in the CQB-competency and hand-hand-combat ratings. Furthermore, *Hardiness* as well as *Comradeship- and Functional Work Attitude* were positively related to the CQB and hand-to-hand combat performance. The second part of the study showed *Hardiness* and *Comradeship and Team Orientation* significantly predicted commando eligibility.

**Discussion:**

Findings from the study support an integrative model describing PCR as a multidimensional, dynamic, and trainable construct. It plays a central role in the basic training and final qualification of special forces personnel. The results reinforce the notion that psychological components such as *Hardiness*, *Functional Work Attitude*, and *Comradeship and Team Orientation* strengthen collective performance and cohesion in high-risk environments. These insights provide empirical evidence for the relevance of psychological factors in military contexts and offer concrete directions for refining selection and training procedures. Overall, the integration of psychological training modules into special forces education proves both meaningful and performance-relevant, positioning PCR as a key resource for enhancing individual preparedness and team effectiveness in complex operational scenarios.

## 1 Introduction

Special forces are highly specialized units deployed in contexts characterized by extraordinary operational stress, significant threats, and exceptional demands on individuals and teams. However varied the concrete tasks of special forces may appear, the requirements in terms of training level and competence are generally significantly higher than in regular forces (1). For some time, it has been widely accepted that, in addition to far above-average physical abilities, a combination of relevant psychological traits is also required for special forces to perform their duties effectively (2).

While the psychological research landscape has so far taken a rather eclectic approach to identifying the significance of individual variables in order to elaborate on their usefulness in the context of special forces, there still appears to be a lack of an integrative model that brings together all the required traits, conceptually relates them to one another, and empirically validates them through appropriate measurement methods.

Psychological Combat Readiness (PCR) is a multidimensional construct that encompasses key psychological attitudes, which enable individuals to maintain high mission commitment and achieve operational success in demanding environments such as military, law enforcement or intelligence community settings (2, 3). This concept was developed through a systematic literature review and expert interviews conducted within German elite tactical units, including the German Army Special Forces Command (Kommando Spezialkräfte, KSK), German Naval Special Forces Command (Kommando Spezialkräfte der Marine, KSM), and the Central Customs Support Unit (Zentrale Unterstützungsgruppe Zoll, ZUZ). The aim was to identify core psychological predictors that contribute to sustained performance under pressure and to integrate them into one overarching model (1, 2). PCR is closely linked to a concept relevant in military psychology, namely *psychological operational readiness* (POR; 2, 4). Psychological readiness Psychological operational readiness is primarily characterized by a group-related positive attitude toward one’s own occupational tasks, high performance, and an absence of psychopathology. In short, individuals are satisfied, capable, and mentally healthy. It is assumed that a broad constellation of traits and contextual factors determines this individual POR. In the context of PCR, it is assumed that these are the individual traits in the special forces setting that predispose individuals to maintain high psychological operational readiness over a prolonged period (1).

PCR is comprised by five central dimensions. The first is *Psychological Stability (PS)*, which refers to a resilient and functional lifestyle shaped by individual circumstances, emotional well-being, and social integration. It is characterized not by the absence of stress, but by the ability to maintain psychological balance and preserve meaningful relationships and activities even under high operational strain. Importantly, this trait is not conceptualized as a fixed personality dimension or as the inverse of neuroticism, but rather as the absence of clinical symptoms and the presence of adaptive life conditions (1, 2).

*Hardiness (H)*, the second dimension, builds on the foundational work of Kobasa (5) and its adaptation for military contexts by Bartone (6). It comprises three interrelated components: *commitment* to one’s role and environment, *challenge* as a mindset that views adversity as an opportunity for growth, and *control*, the perceived ability to influence events in both personal and professional domains. Together, these elements foster psychological resistance and adaptability in high-stakes scenarios (7).

The third dimension, *Functional Work Attitude (FA)*, describes an intrinsically motivated and performance-oriented approach to professional duties. Individuals who score high in this competence demonstrate reliability, discipline, and conscientiousness. They pursue high-quality outcomes and remain receptive to constructive feedback, all while maintaining a pragmatic and goal-directed mindset. Their motivation is internally driven, allowing them to operate effectively without reliance on external incentives (1, 2).

The fourth psychological component, *Comradeship and Team Orientation (CTO)*, reflects a stable disposition toward supporting the social structure within operational units. Individuals with strong team orientation actively contribute to group cohesion, integrate seamlessly into hierarchical structures, and prioritize collective success over personal interests. Their interpersonal behavior is marked by professional reliability, mutual respect, and collegial care—even in emotionally charged or private matters. This attitude plays a critical role in fostering *Military Unit Cohesion*, as conceptualized by Siebold (8), and is essential for maintaining functional group dynamics under high stress situations (1, 2).

Finally, *VUCA Competence* refers to the ability to adapt effectively to volatile, uncertain, complex, and ambiguous operational environments (in short, VUCA). This trait has gained prominence through research on crisis deployments and reflects a unique capacity to remain flexible, situationally aware, and decision-capable in rapidly changing contexts (9, 10).

Together, these five PCR-traits form a comprehensive framework for understanding and assessing psychological readiness in high-demand operational roles. PCR offers a valuable conceptual foundation for personnel selection, training, and resilience-building in environments where psychological performance is critical to mission success.

### 1.1 Scientific background of psychological combat readiness and alternate approaches

Since the annexation of Crimea by Russia in 2014, the combat readiness of military units—and particularly the physical and psychological performance of individual soldiers—has regained strategic importance within the German Armed Forces Bundeswehr. This renewed focus is reflected in the concept of a “mindset for national and alliance defense” (11). Central to this framework are psychological factors such as resilience, discipline, motivation, performance orientation, and both physical and mental endurance (11).

In the development of a military readiness questionnaire, Wen et al. (12) emphasized the importance of psychological attitudes for combat readiness within military units. Key elements include unit cohesion, comradeship, discipline, motivation, willingness to engage, and trust in leadership. These findings were empirically supported by Griffith (13) and Handayani and Widana (14), who demonstrated that psychological well-being, resilience, and leadership style have a positive impact on unit cohesion and mission readiness.

Building on the recognition that psychological components are essential to military readiness, Michels & Mair (2) introduced the concept of *Psychological Combat Readiness (PCR)*. PCR refers to the psychological characteristics at the individual level that can support soldiers in maintaining and enhancing their performance during combat and crisis situations over extended periods. Distinct from conventional resilience frameworks, PCR incorporates a multidimensional perspective that goes beyond stress management and the maintenance of mental health (15–19). While the integrative nature of the PCR framework is new, there has been extensive research in regards to its incorporated concepts.

PCR differs by focusing specifically on high-performing soldiers who already exhibit strong resilience, motivation, and a pronounced military commitment (20). Studies show that PCR is particularly prevalent among special operations forces and specialized units within the Bundeswehr (1–3).

*Psychological Stability (PS)* is a functional construct defined by current life circumstances, lifestyle, and mental well-being. It is not a fixed personality trait but reflects the absence of psychopathological symptoms. Low *PS* does not necessarily indicate clinical disorders but may signal increased risk for emotional and behavioral dysregulation. Indicators of high PS include social integration, life satisfaction, and meaningful engagement (2, 3).

Moreover, *PS* describes a functional, performance-enhancing lifestyle and environment, and should therefore be distinguished from Antonovsky’s concept of Sense of Coherence (21), which refers more to a general attitude and cognitive style aimed at creating meaningfulness and resources within life circumstances.

In contrast to emotional stability in the Big Five model, *PS* is a performance-oriented concept. Military psychology studies show that a psychological functional lifestyle contributes to decision-making, stress management, and operational persistence under pressure (22–24). Soldiers with high *PS* demonstrate stable performance and potentially have a lower risk of psychological disorders (1).

*PS* also strengthens team dynamics by fostering trust and cohesion under stress (25). Empirical evidence links *PS* to reduced PTSD risk, lower burnout rates, and successful completion of military training programs (3, 26–28).

*Hardiness (H)* has emerged as a robust predictor of psychological resilience and combat readiness in military contexts. Skomorovsky and Sudom (29) found that *Hardiness* positively influences mental health among Canadian officers. Bartone (6) described “hardy individuals” as those who experience lower psychological strain following stressful life events and possess greater resources and more effective coping mechanisms (5, 30).

Beyond its health-preserving effects, *Hardiness* has been linked to improved operational performance. Giatras (31) and Bartone (23) demonstrated that firefighters and U.S. soldiers with higher *Hardiness* scores performed better in mission-specific scenarios and exhibited enhanced cognitive functioning. Lo Bue et al. (32) found that recruits with higher *Hardiness* levels achieved significantly better performance results in close-combat training. Furthermore, Pattyn et al. (33) showed a tendency for soldiers with high *Hardiness* to perform better in the Belgium special forces selection and Bekesiene et al. (2022) (34) showed significant results for reserve soldiers with high *Hardiness* scores on tactical and military performance.

Importantly, *Hardiness* is not innate but trainable. Khoshaba and Maddi (35) developed a training program that has since been integrated into PCR training modules (1, 36). Training outcomes include improved performance, increased health awareness, and post-traumatic growth. Maddi (37) also showed that hardy individuals are more resistant to extremist influences, underscoring their emotional stability and character strength. The hardiness training module of the presented studies includes:

1. Enhancing interpersonal and conflict resolution skills (38)
2. Reframing critical life events as challenges – cognitive reframing and reappraisal (39)
3. Strengthening perceived control through “Locus of Control” shifts (40, 41) in both professional and personal contexts (2)

*Functional Work Attitude (FA)* is the third core component of PCR. Special forces require high performance motivation and a continuous drive for improvement to operate effectively under extreme stress (42). Research shows that SOF operators possess a particularly strong performance orientation and adaptability (20). They must be receptive to feedback and capable of analyzing and optimizing their skills. Self-regulation and emotional control are essential for managing complex operational scenarios and high stress situations (43).

A strong *FA* is associated with high self-regulation, emotional control, and learning readiness. The ability to improve continuously and embrace new challenges distinguishes elite soldiers from regular infantry (1, 3, 20, 44). Optimizing these abilities enhances both individual and unit performance. Training in *FA* and performance motivation should include strategies for self-regulation, emotional management, and learning agility (2).

*Comradeship and Team Orientation (CTO)* are critical to PCR, especially at the individual level. Siebold (8) defines unit cohesion as the bond within military groups formed through social relationships and trust. Primary cohesion refers to direct interactions within platoons and between peers and leaders, while secondary cohesion relates to broader organizational levels such as companies or brigades (45). Effective unit cohesion is essential for PCR, as it arises from individual commitment to team dynamics. It involves not only perceiving social cohesion but actively contributing to it (2). For special forces, primary cohesion is particularly vital due to its direct impact on performance under extreme conditions (46). Studies show that perceived group bonding and comradeship significantly enhance combat effectiveness and readiness (12, 47, 48), while also improving psychological well-being, reducing stress, and strengthening identification with military service (13, 48, 49).

High cohesion also boosts morale before, during, and after deployments (50). Furthermore, Williams et al. (51) showed cohesion during basic training improves marksmanship and unit resilience. Ben-Shalom et al. (46) confirmed that team cohesion positively influences performance, especially in small units. Enhancing team orientation in the PCR-training involves improving soldiers’ social and communication skills (52, 53), with effective communication being a prerequisite for primary cohesion (8).

Modern operational environments are increasingly shaped by volatility, uncertainty, complexity, and ambiguity—summarized under the acronym *VUCA* (54). Effective performance in such conditions requires *VUCA competence*: the ability to remain operationally capable despite unpredictable and ambiguous scenarios (55, 56).

VUCA competence includes cognitive problem-solving, emotional resilience, and patent acceptance of uncertainty. It emphasizes decision-making under limited resources and incomplete information, viewing uncertainty as a normal part of professional action (2, 3).

Originally developed by the U.S. War College in the 1990s, the VUCA framework has since been adopted across sectors (57). In military contexts—especially SOF—*VUCA competence* is a key leadership resource (9, 57). Studies show that it correlates with improved cognitive performance, decision quality, and reduced burnout risk (33, 58, 59).

Military selection processes increasingly assess *VUCA* traits through stress-based simulations and decision-making exercises, such as those used in the German Army Special Forces selection process (26) and basic SOF training (1). Initial findings suggest that individuals with high *VUCA competence* are more likely to succeed in military careers (33, 57, 60). Although the concept of *VUCA* has gained prominence in military and organizational psychology, few empirical studies have successfully operationalized it as a trainable construct (9). Its multidimensional nature and context-dependent manifestations pose significant challenges for standardized training interventions. Given these limitations, we deliberately chose to delegate the *VUCA* module to our experienced military training NCOs, who are best positioned to address situational adaptability through field-based instruction and scenario-driven learning.

Instead, our research focus was directed toward psychological modules with clearer theoretical foundations and established measurement frameworks: *Hardiness, Comradeship and Team Orientation, and Functional Work Attitude.* These constructs are not only empirically linked to performance under stress and cohesion in high-risk environments, but also lend themselves to structured training and longitudinal evaluation. By concentrating on these dimensions, we aimed to enhance individual *Hardiness*, interpersonal effectiveness, and mission-oriented mindset—core attributes that indirectly support *VUCA*-readiness through foundational psychological strength.

### 1.2 Research question

To systematically investigate the relevance of Psychological Combat Readiness (PCR) on the performance of special forces candidates, a two-part longitudinal study design was developed. This framework spans the entire selection and training process, from the initial potential assessment to the final qualification as a Commando soldier. The central assumption guiding this research is that PCR plays a critical role in basic SOF training (study 1) and in the commando qualification (study 2) of the German Army Special Forces.

The primary objective was to analyze whether and to what extent PCR predicts performance in different phases of the training process and the successful completion of the Commando qualification pathway.

The first intervention study examined the effects of a structured psychological training program designed to enhance PCR on military and tactical performance during basic SOF training. Specifically, it assessed whether targeted psychological interventions could positively influence *Hardiness*, *Functional Work Attitude*, *Comradeship and Team Orientation*, and *Operational Effectiveness*.

Study 2 evaluated the relationship between PCR as a predictor for successful completion of advanced Commando qualification and the award of the commando badge.

**Figure 1:**
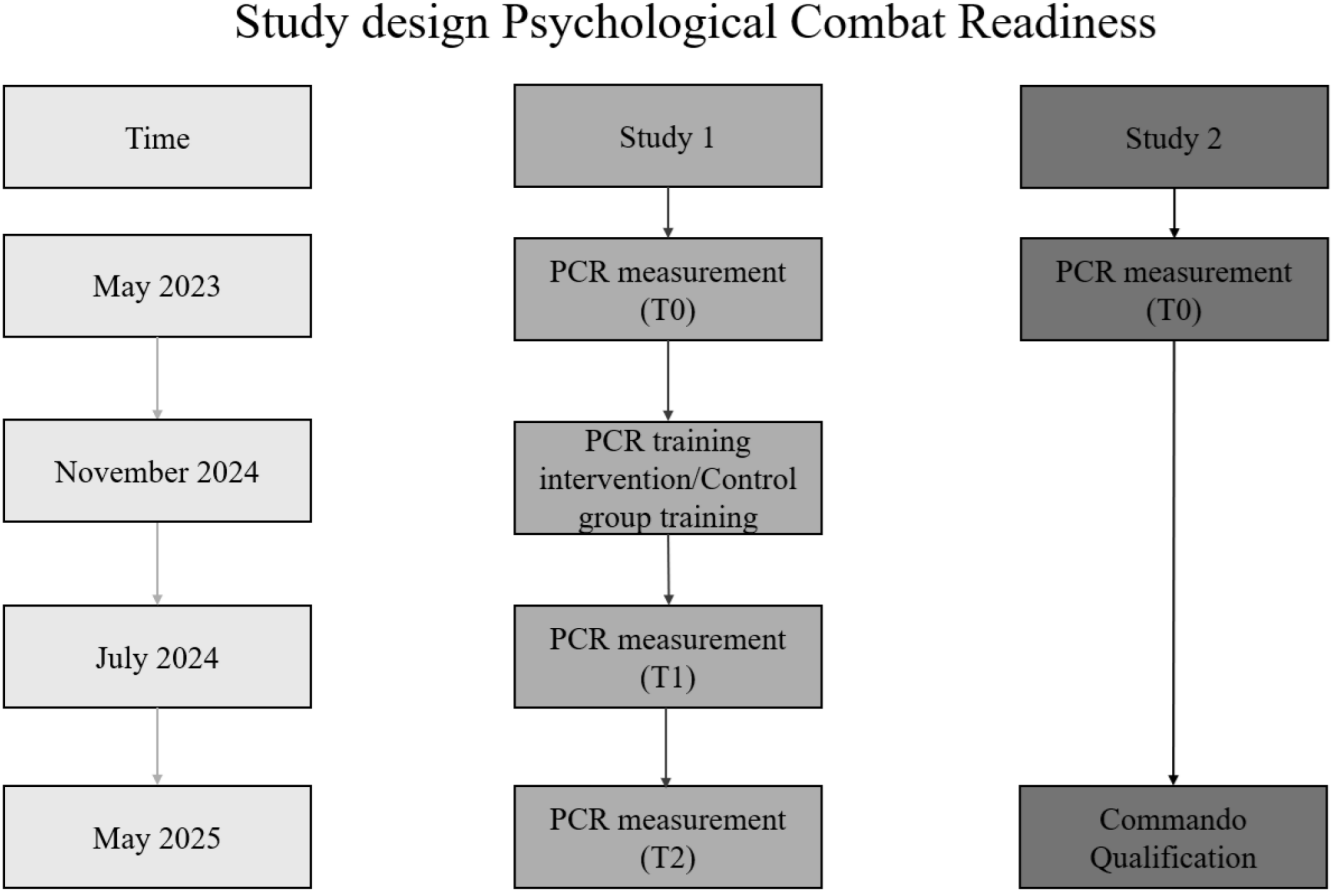
Study design and measurements of psychological combat readiness. Describes the study design and points of measurement of Psychological Combat Readiness.

Together, these studies address the following overarching research questions and are presented in **Figure 1**:

1. *Does the Psychological Combat Readiness have a measurable impact on military and tactical performance during training?*
2. *Can Psychological Combat Readiness be considered a valid predictor of Commando Qualification?*

For Study 1, the primary outcome is the longitudinal change in Psychological Combat Readiness (PCR) across the three measurement time points (baseline, 15 months, 24 months), operationalized through its five dimensions (Hardiness, Functional Work Attitude, Comradeship & Team Orientation, Psychological Stability, VUCA Competence). Military‑tactical indicators such as Close Quarter Battles (CQB), hand‑to‑hand combat, and advanced CQB performance serve as secondary outcomes. The primary objective of Study 1 is to examine whether the PCR‑training intervention leads to significant improvements in the PCR dimensions compared with the control group. For Study 2, the primary outcome is the binary Commando Qualification result after 24 months (qualified vs. not qualified). The primary objective of Study 2 is to determine whether baseline PCR dimensions predict successful qualification.

## 2 Methods

Due to operational confidentiality and personnel security requirements within the German Army Special Forces Command (KSK), no specific information regarding sample size or cohort composition is disclosed. This measure protects the operational capabilities and safety of deployed personnel. Disclosure of such details could compromise current or future missions and is therefore subject to strict classification protocols.

### 2.1 Sample description

Participants in this study were active Commando candidates undergoing basic SOF training within the German Army Special Forces Command (KSK) between 2023 and 2025. Group assignment into the experimental group (EG) and control group (CG) was determined based on existing training schedules and logistical constraints. For operational and instructional reasons, candidates could not be randomly distributed across groups, as this would have disrupted established military and training continuity. The integrity of pre-existing group cohesion—essential for tactical instruction and mission preparation—was preserved throughout the intervention period. Although not randomized, both groups were comparable in terms of training phase, demographic composition, and baseline psychological readiness.

### 2.2 Measures

The Psychological Combat Readiness Questionnaire (German: Psychologische Einsatzfähigkeit) was conceptualized and standardized by Michels and Mair (see 1–3) for special operations settings. It contains 50 items measuring five areas: *Hardiness, Psychological Stability, VUCA Competence, Functional Work Attitude, and Comradeship and Team Orientation*, representing the concept of PCR primarily for military and law enforcement roles. In Studies 1 and 2 Cronbach’s Alpha values for the five subdimensions of Psychological Combat Readiness were as follows: Hardiness (α = .82), Psychological Stability (α = .79), VUCA Competence (α = .76), Functional Work Attitude (α = .84), and Comradeship & Team Orientation (α = .87). Another study from Mair et al. (61, in print) reported similar internal consistency values. The survey was conducted voluntarily, with participants rating their agreement with each item on a 5-point Likert scale ranging from 1 (“strongly disagree”) to 5 (“strongly agree”).

The PCR questionnaire comprises multiple subdimensions, each reflecting a core component of psychological readiness. For the purpose of this study, the following dimensions were analyzed:

The dimension *Hardines*s reflects psychological persistence and stress tolerance. It is separated into the three domains control, commitment and challenges. Example items are: *“I see difficult situations not as problems, but as interesting challenges”* and *“Tough experiences have helped me grow.”*

*Functional Work Attitude* measures goal orientation and intrinsic motivation for duty. Example items are: *“My superiors often need to motivate me to perform my duties”* (inverted) and *“I ask my teammates for feedback or criticism about my performance.”*

*Comradeship and Team Orientation* assess interpersonal attitude to cohesion and reliability within teams. Example items are: *“My teammates can always rely on me”* and *“I feel a* sp*ecial bond with my team.”*

*Psychological Stability* captures emotion regulation and a functional lifestyle. Example items are: *“My family and friends provide strong support”* and *“Military service is the only meaningful thing in my life”* (inverted).

*VUCA Competence* refers to the ability to operate effectively in volatile, uncertain, complex, and ambiguous environments. Example items are: *“I can tolerate unpredictable, unstructured situations well”* and *“I find it exciting to face complex, unclear situations.”*

### 2.3 Military performance ratings

To assess the military and tactical performance of the commando candidates, an external performance evaluation was incorporated as a key criterion. Specifically, shooting proficiency in Close Quarter Battle (CQB) (62) scenarios was rated weekly by a panel of eight instructors—four internal and four external experts. Ratings were assigned on a five-point Likert scale, ranging from 1 (“does not master CQB”) to 5 (“fully masters CQB and all tactical components”). At the conclusion of the training cycle, the individual weekly scores were averaged to yield a composite measure of each candidate’s tactical performance throughout the course and averaged at the end of the course.

In addition to CQB performance, military proficiency in tactical hand-to-hand combat was systematically assessed. A comprehensive test battery comprising over ten physical performance criteria—including punching power, explosive strength, speed, coordination, and muscular endurance—was administered. Each candidate of the experimental and control group could achieve a total score ranging from 0 to 100 points. Based on these results, a performance ranking was established across all participants. Furthermore, various mission-relevant sparring scenarios were technically and tactically evaluated, and an expert panel of instructors was convened to provide a final performance appraisal. At the end of the training phase, each candidate received a final ranking and an overall score on a 0–100 scale, reflecting their tactical combat capabilities.

Finally, military and tactical performance during advanced Close Quarter Battle (CQB) scenarios were assessed in a transitional evaluation phase. At the end of the training cycle, candidates were classified as either successful (1) or unsuccessful (0), with the latter category including both those who failed to meet the required standards and those who were withdrawn from the course. This binary classification was based on performance in high-intensity CQB exercises designed to simulate operational conditions. The German Army Special Forces Command utilized a standardized evaluation framework to assess tactical proficiency and decision-making under pressure. Due to operational security constraints, the specific criteria used in this framework cannot be publicly disclosed. Nevertheless, the classification system provided a robust endpoint measure of tactical readiness and allowed for comparative analysis across candidates.

### 2.4 Study 1: psychological combat readiness as intervention and military performance indicator

Study 1 was conducted between 2023 and 2025 during the basic SOF training phase for Commando candidates. Participants were divided into two groups: an experimental group (EG) and a control group (CG), following a quasi-experimental design. The experimental group took part in a structured Psychological Combat Readiness (PCR) training program, while the control group engaged in relaxation-based interventions, including progressive muscle relaxation and breathing techniques. PCR training for the experimental group occurred on three working days within an organizational week during basic SOF training. In contrast, the control group received three half-day relaxation-based intervention sessions, scheduled four weeks later (compare **Figure 1** and **Table 1**). The EG and CG were unaware of the specific training or conditions they experienced, as the study was presented as psychological performance training. While group assignment preserved existing training continuity, it is possible that candidates from the experimental and control groups interacted during overlapping training activities. Although the study was presented as general psychological performance training and the specific content of interventions was not disclosed to participants, we cannot fully exclude the possibility of informal discussions between groups regarding training content

**Table 1:** Overview of study design and measurement timepoints.

Study	Timepoint	Group	Intervention	Measurement/Outcome
Study 1	May 2023 (Baseline) Training November 2023	EG & CG	EG: PCR-training (3 days) CG: Relaxation-based training (3 half days, 4 weeks later)	PCR (all dimensions)
Study 1	August 2024 (15 months)	EG & CG	No intervention	PCR reassessment CQB, hand-to-hand combat, tactical evaluation
Study 1	May 2025 (24 months)	EG & CG	No intervention	PCR reassessment advanced CQB
Study 2	May 2025	Full cohort	No intervention	Qualification outcomes (1 = qualified, 0 = not qualified; Logistic regression using PCR baseline predictors

PCR was reassessed at 15 and 24 months. Military and tactical performance were also evaluated using standardized measures such as Close Quarter Battle (CQB) proficiency, advanced CQB assessment and tactical hand-to-hand combat capability.

### 2.5 Training material psychological combat readiness

The PCR training was divided into three modules:

1. Hardiness – based on frameworks from Khoshaba and Maddi (35) Reivich et al. (63), and the Canadian Special Forces SOMA program (64), focusing on problem-solving, mental agility, cognitive reframing, control beliefs, service commitment, and character strengths (65, 66).
2. Comradeship and Team Orientation – emphasizing interpersonal communication (38), leadership under stress (67), and emotional regulation, drawing on models such as Nonviolent Communication (68–70), Active Constructive Responding (71), and emotion regulation strategies (68, 72).
3. Performance Optimization – including self-monitoring, activation management, visualization strategies and goal-setting strategies adapted from the Human Performance Systems domain of the German Army Special Forces Command and psychology performance protocols (1, 64, 73, 74)

A comprehensive review of the training materials is provided in Mair et al. (1), which offers an in-depth analysis of the PCR training resources.

### 2.6 Study 2: psychological combat readiness as a predictor for commando qualification

In May 2025, half of the candidates successfully completed the training and were awarded the Commando badge. The PCR scores recorded at the beginning of the basic SOF training in May 2023 were compared with those of individuals who subsequently achieved Commando qualification (compare **Figure 1** and **Table 1**). A binary logistic regression was conducted using the five PCR dimensions—as predictors of qualification outcome (coded as 1 = qualified, 0 = not qualified). Furthermore, the results comparing the experimental and control groups regarding success rates in commando qualification across the whole study sample were evaluated.

### 2.7 Statistical analysis

All statistical analyses were conducted using Python (version 3.12), the statsmodels and scikit-learn libraries and Microsoft Excel advanced statistic package. To evaluate the predictive value of PCR-factors on military performance and qualification outcomes, a series of binary logistic regression models and a Chi-Square Test were computed.

To examine the psychological and performance-related effects of the training intervention and commando qualification predictability, a series of statistical models were specified and evaluated. Each logistic regression model included psychological predictors—namely *Hardiness (H)*, *Functional Work Attitude (FA)*, and *Comradeship & Team Orientation (CTO)—*as independent variables, alongside group assignment (Experimental vs. Control). Interaction terms between predictors and group were incorporated to assess differential effects across conditions.

Prior to analysis, all continuous predictors were z-standardized to ensure comparability and facilitate interpretation of regression coefficients. For each model, Wald test statistics (z-values and p-values) were reported for individual predictors, along with Odds Ratios (OR) and 95% Confidence Intervals (CI), derived via exponentiation of the regression coefficients. Model fit was evaluated using McFadden’s R² and Nagelkerke’s R², the latter scaled to a [0, 1] range for comparability with linear models.

Separate models were computed for each psychological predictor, followed by a combined model that included all predictors and their respective interaction terms. Improvements in model fit were assessed by examining changes in Nagelkerke’s R² across models.

To evaluate long-term effects of the training program, independent-samples t-tests and ANOVAs were conducted to compare PCR scores between groups at multiple time points. Effect sizes were calculated using Cohen’s d and partial eta squared (η^2^_p_).

Additionally, rMANOVA were performed to investigate group differences across multiple Psychological Combat Readiness (PCR) factors and performance indicators. Interaction effects were tested to explore whether patterns varied between the experimental and the control group.

All statistical tests were one-tailed, based on directional hypotheses derived from theoretical and empirical expectations. Statistical significance was set at p <.05. Marginal effects (p <.10) were interpreted with caution and discussed in the context of exploratory analysis.

### 2.8 Ethical considerations

All participants were informed about the study and provided written consent. PCR assessments were administered prior to tactical combat training sessions, before Close Quarter Battle training and during administration week with a standardized 10-minute completion window.

This study was conducted in accordance with the ethical standards of the University of the Bundeswehr Munich. The research protocol was reviewed and approved by the university’s ethics committee. All procedures involving human participants were carried out in line with institutional guidelines and the principles of the Declaration of Helsinki. Informed consent was obtained from all participants prior to their inclusion in the study. Participation in the study had no negative consequences for the participants’ military careers or their eligibility for later commando positions.

## 3 Results

Due to operational security protocols of the German Army Special Forces Command (KSK), specific details regarding sample sizes, degrees of freedom, and unit-level identifiers cannot be disclosed. This restriction ensures the protection of personnel identities, mission-sensitive structures, and internal organizational configurations. Therefore, in study 1 a traditional *a priori* power analysis was not feasible. To ensure methodological adequacy, a sensitivity analysis was conducted for the repeated-measures MANOVA and the related follow-up tests. This analysis indicated that the available sample provided sufficient power (α = .05, power = .80) to detect small-to-moderate multivariate effects (minimum detectable partial η² of approximately.04–.06) and moderate univariate effects in the t-tests (minimum detectable Cohen’s d ≈ 0.45–0.55). To nevertheless ensure the methodological adequacy of the logistic regression models in study 2, a sensitivity analysis was conducted using the available sample and the observed distribution of successful versus unsuccessful candidates. This analysis indicated that the study was sufficiently powered to detect moderate-to-large effects, corresponding to minimum detectable odds ratios of approximately 2.5–3.0 at α = .05 and power = .80. Thus, the model was adequately equipped to identify psychologically meaningful associations between PCR factors and qualification success, within the constraints of the operational context. All statistical analyses were conducted in accordance with ethical standards and methodological rigor, while respecting the confidentiality requirements of the participating military unit.

### 3.1 Study 1: psychological combat readiness as intervention and military performance indicator

In the first study, PCR training demonstrated measurable effects on key psychological constructs, including *Hardiness (H*), *Functional Work Attitude (FA)*, and self-reported *Performance Motivation* from the start of basic SOF training to the 15-month assessment point. Box’s M indicated a significant deviation from the homogeneity of covariance matrices (*p*= .026). Due to this violation and the well-documented robustness of Pillai’s Trace under such conditions, Pillai’s Trace was selected as the primary test statistic for the repeated-measures MANOVA (rMANOVA). The rMANOVA revealed a marginal group effect (F = 1.91, *p*= .17, η^2^_p_ = .01; Pillai’s Trace = .004), significant main effects of PCR factors on military performance (F = 222.37, *p*< .001, η^2^_p_ > .14; Pillai’s Trace = .277), and a significant interaction between group and PCR factors (F = 2.80, *p* < .001, η^2^_p_ = .11; Pillai’s Trace = .082), indicating that the intervention differentially influenced psychological constructs over time.

**Figure 2:**
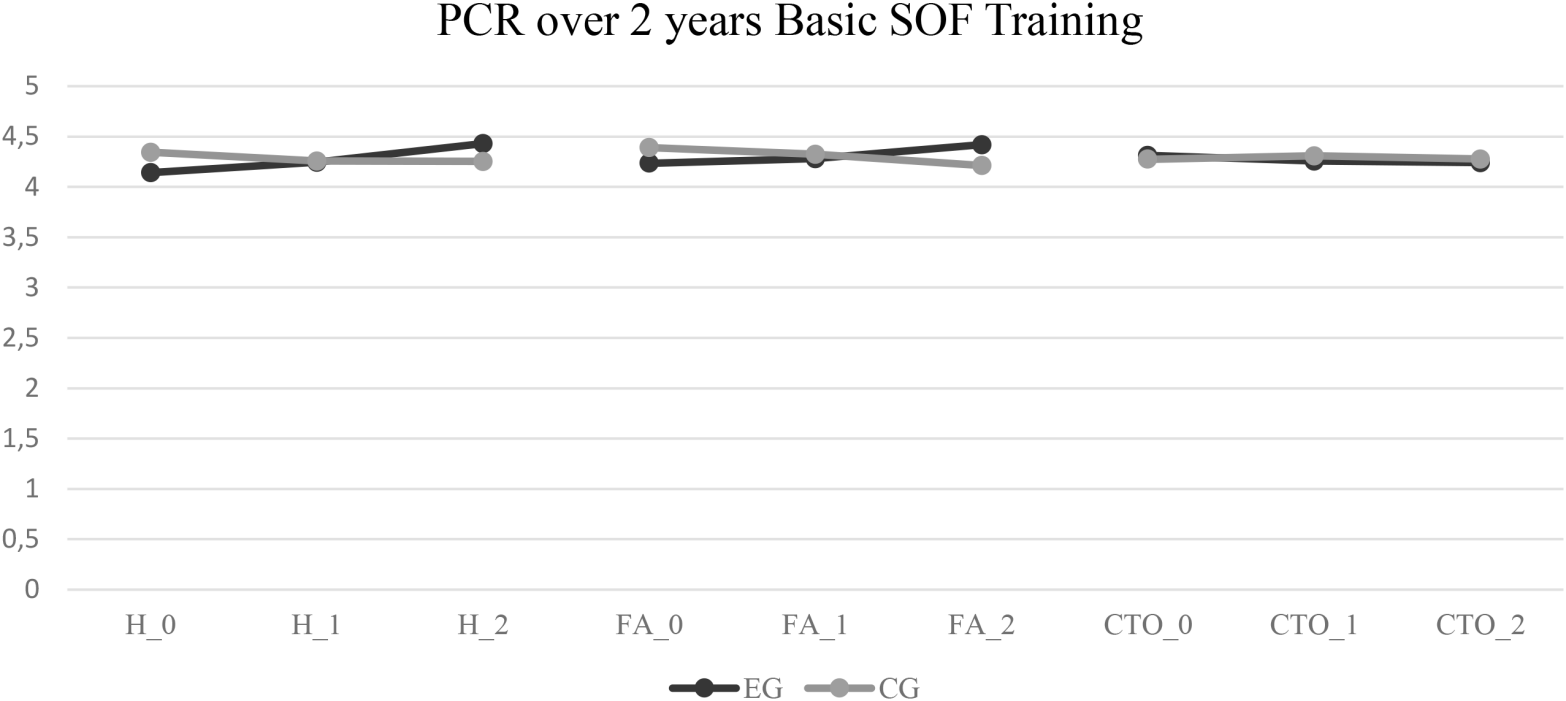
Longitudinal comparison of psychological combat readiness factors in the basic SOF training of the KSK. Describes the changes of Psychological Combat Readiness factors Hardiness (H), Functional Work Attitude (FA) and Comradeship and Team Orientation (CTO) between the starting point of the Basic SOF training (t0), after 15 months (t1) and after 24 months (t2) between the experimental group (EG) and control group (CG).

Independent t-tests at the final assessment point (May 2025) initially suggested significant group differences in *Hardiness* (t = 2.02, *p* = .04, *d* = 0.53) and *FA* (t = 2.29, *p*= .02, *d* = 0.61), favoring the intervention group, while no difference was observed for *CTO* (*p* = .58). However, because multiple group comparisons were conducted across all timepoints and performance outcomes, Benjamini–Hochberg false-discovery rate (FDR) corrections were applied to control for the increased Type I error rate. After FDR adjustment, none of these mean-level differences remained statistically significant, including those that were initially significant or marginal at the unadjusted level. Unadjusted p-values are reported for transparency. **Figure 2** provides an overview of the descriptive changes in *Hardiness* and *Functional Work Attitude* across the three assessment periods (baseline, 15 months, and 24 months).

At the onset of basic SOF training, small group differences in PCR scores were observed. A trend-level difference emerged in *Hardiness* (EG: *M* = 4.14, *SD* = 0.45; CG: *M* = 4.34, *SD* = 0.31; t = –1.43, *p* = .08), and *FA* showed a statistically significant unadjusted difference (EG: M = 4.24, *SD* = 0.17; CG: *M* = 4.39, *SD* = 0.28; t = –1.41, *p* =.041). After FDR correction, these baseline differences were no longer statistically significant.

**Figure 3:**
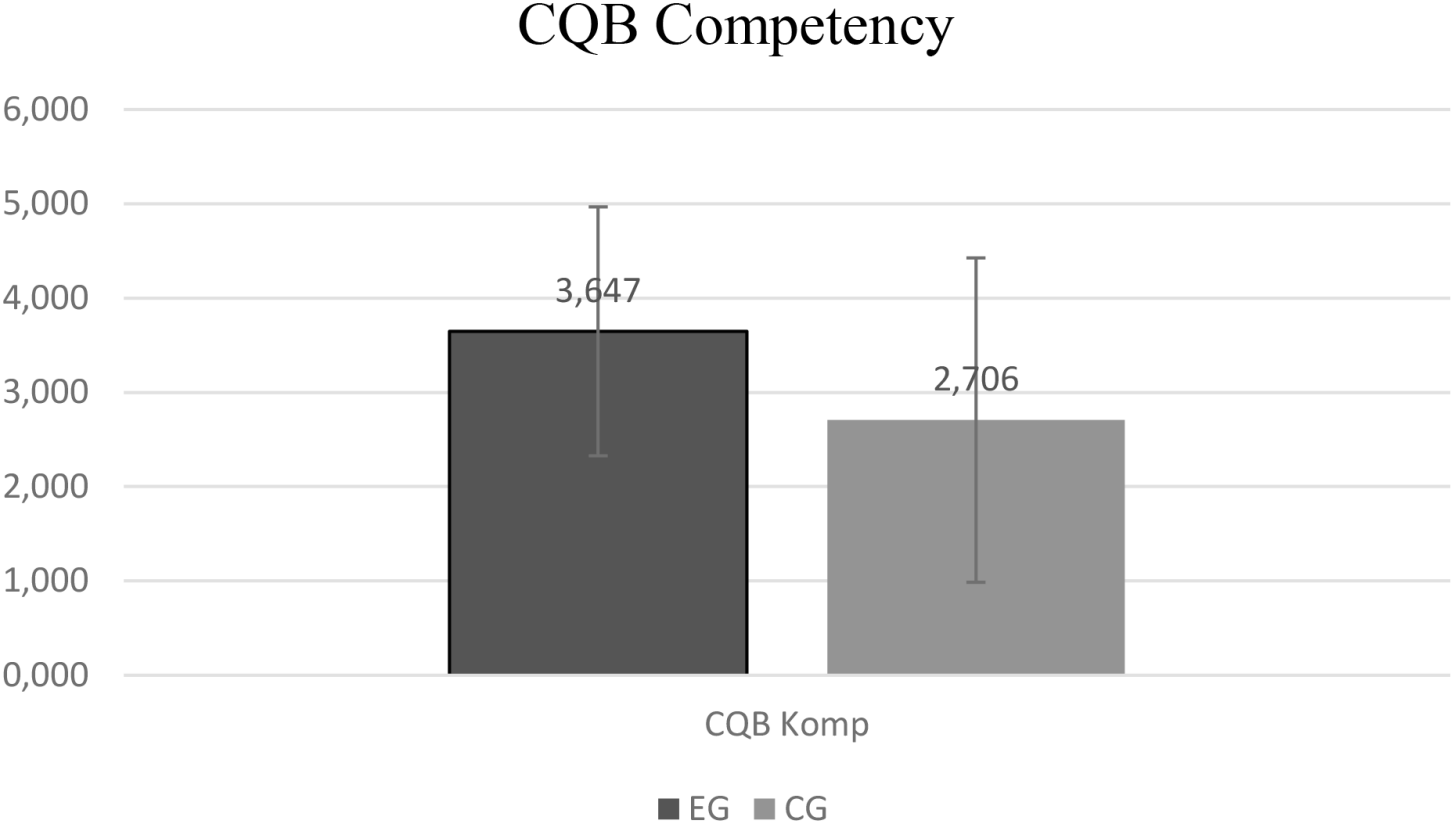
Averaged comparison of Close Quarter Battle (CQB) performance ratings after 15 months. Describes the differences in military and tactical performance based on competency ratings between the experimental and control groups in CQB.

Regarding military performance outcomes, unadjusted analyses indicated that the experimental group performed better in the Close Quarters Battle (CQB) course after 15 months (EG: *M* = 3.65, *SD* = 0.34; CG: *M* =2.71, *SD* = 0.41; t = –1.85, *p* = .037, *d* = 0.60). Marginal differences were observed in hand-to-hand combat training (*p* = .06) and self-assessed performance motivation (*p* = .07). None of these performance-related comparisons survived FDR correction, and they should therefore be interpreted with caution. **Figure 3** visualizes the descriptive performance differences.

**Figure 4:**
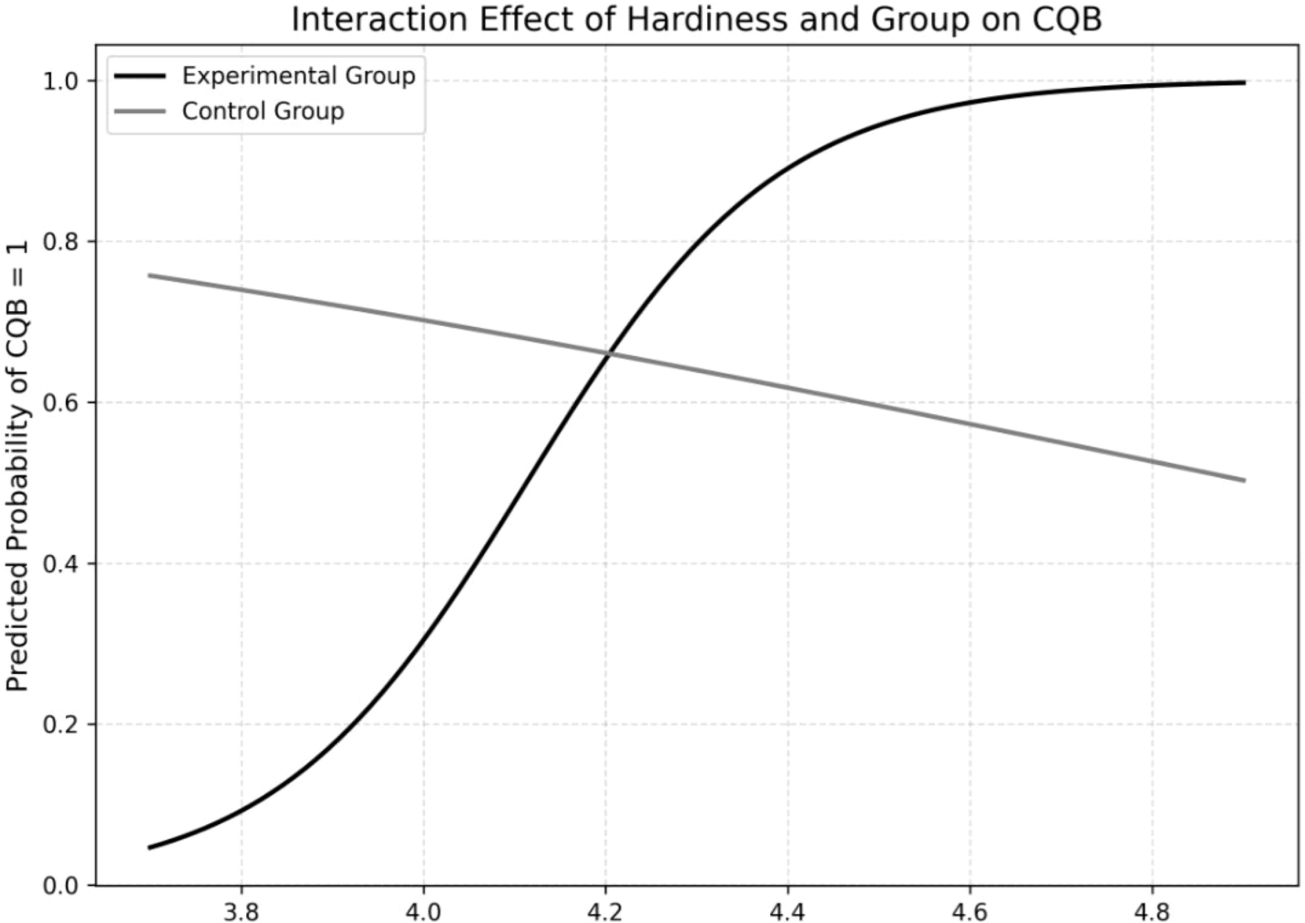
Prediction of hardiness for passing advanced Close Quarter Battle (CQB) after 24 months. Indicates that higher Hardiness scores are more likely in the experimental group than the control group for passing advanced CQB.

**Figure 5:**
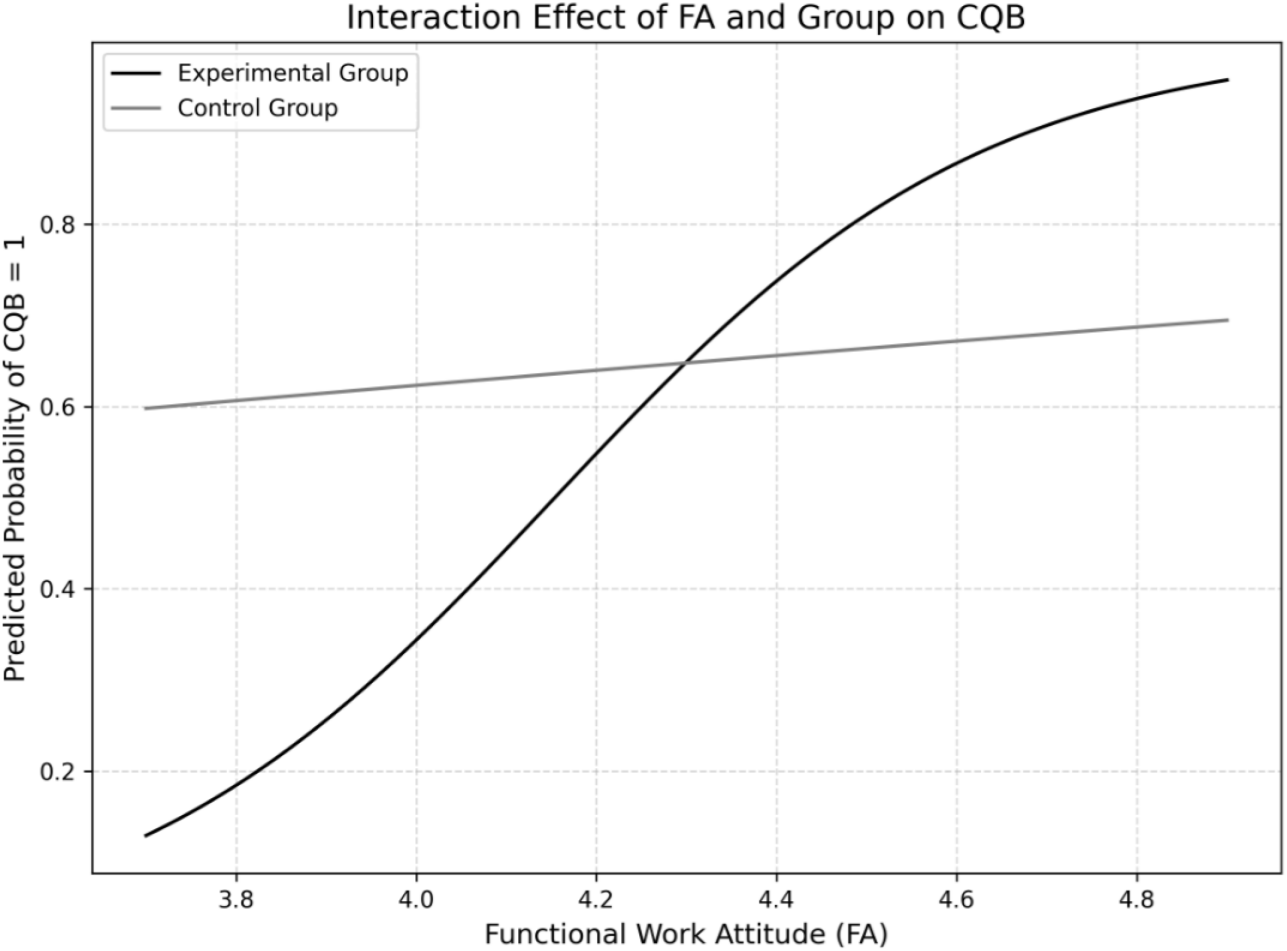
Prediction of functional work attitude for passing advanced Close Quarter Battle (CQB) after 24 months. Indicates the tendency that higher FA scores are more likely in the experimental group than the control group for passing advanced CQB.

The advanced Close Quarter Battle Course (aCQB), completed at the end of the 24-month training program, was analyzed using a Chi-Square Test, revealing a marginal difference (χ² = 2.124, *p* = .08). Logistic regression models including interaction effects (Hardiness × Group × aCQB; FA × Group × aCQB) showed that Hardiness was a significant predictor of successful aCQB completion within the experimental group (OR = 13.10, *p* = .047), explaining 34% of model fit (Nagelkerke-R² = 0.3395). *FA* showed a directional but non-significant effect (OR = 3.47, *p* = .08; Nagelkerke-R² = 0.1359). **Figures 4**, **5** illustrate the relationships between *Hardiness*, *FA*, and aCQB success. Full regression results are listed in **Table 2** and **Table 3**. The three remaining PCR components showed no significant effects and were excluded from the final model.

**Table 2:** Predictor of hardiness x group x advanced CQB after 24 months.

Predictor	β coefficient	P-values	Odds-ratio	95 %-CI (OR)
H x EG	+7.30	.047*	13.10	[1.03, 166.36]
Group	-0.59	.570	0.55	[0.07, 4.27]
Intercept	+2.60	.174	3.28	[0.59, 18.21]
H x CG	-2.85	.037*	0.055	[0.004, 0.837]

H, Hardiness; EG, Experimental Group; CG, Control Group; OR, Odds Ratio; 95% CI, Confidence Interval for the Odds Ratio, Significance level: p <.05 (*).

**Table 3:** Predictor of functional work attitude x group x advanced CQB after 24 months.

Predictor	β coefficient	P-values	Odds-ratio	95 %-CI (OR)
FA x EG	+4.20	.137	3.47	[0.67, 17.90]
Group	-0.005	.995	1.00	[0.19, 5.10]
Intercept	+0.61	.312	1.85	[0.56, 6.09]
FA x CG	-1.12	.248	0.32	[0.05, 2.21]

FA, Functional Work Attitude; EG, Experimental Group; CG, Control Group; OR, Odds Ratio; 95% CI, Confidence Interval for the Odds Ratio, Significance level: p <.05 (*).

### 3.2 Study 2: psychological combat readiness as a predictor for commando qualification

The second study examined whether Psychological Combat Readiness (PCR) components predicted successful completion of the 24-month basic SOF training required for special forces qualification. Consistent with Study 1, *Hardiness* and *CTO* emerged as central psychological factors associated with qualification outcomes. Soldiers who scored higher on these PCR dimensions demonstrated a substantially greater likelihood of completing the demanding training pipeline and qualifying as special operations personnel.

A sensitivity analysis of the logistic regression model indicated that, given the available sample size and the distribution of successful versus unsuccessful candidates, the analysis was sufficiently powered to detect moderate-to-large effects (minimum detectable odds ratios of approximately 2.5–3.0 or higher). The final logistic regression model, which included five PCR predictors, significantly explained variation in commando qualification outcomes (χ²(5) = 16.91, *p* < .001). Approximately 41% of the variance in qualification was accounted for by PCR factors (Nagelkerke’s R² = .41; McFadden’s R² = .33). Model fit indices further supported this interpretation (log-likelihood = −20.40; BIC = 52.00), suggesting reasonably good discriminatory value while avoiding model overfitting.

Two predictors showed statistically significant associations with qualification success. *Hardiness* was a robust predictor (β = 1.52, *p*= .012; OR ≈ 4.58), indicating that each one-unit increase in *Hardiness* was associated with more than a threefold increase in the odds of completing the training. In addition, *CTO* demonstrated a meaningful and statistically significant effect (β = 0.98, *p*= .034; OR ≈ 2.66), highlighting the relevance of interpersonal cohesion and team-oriented behavior for success in high-performance military environments.

**Figure 6:**
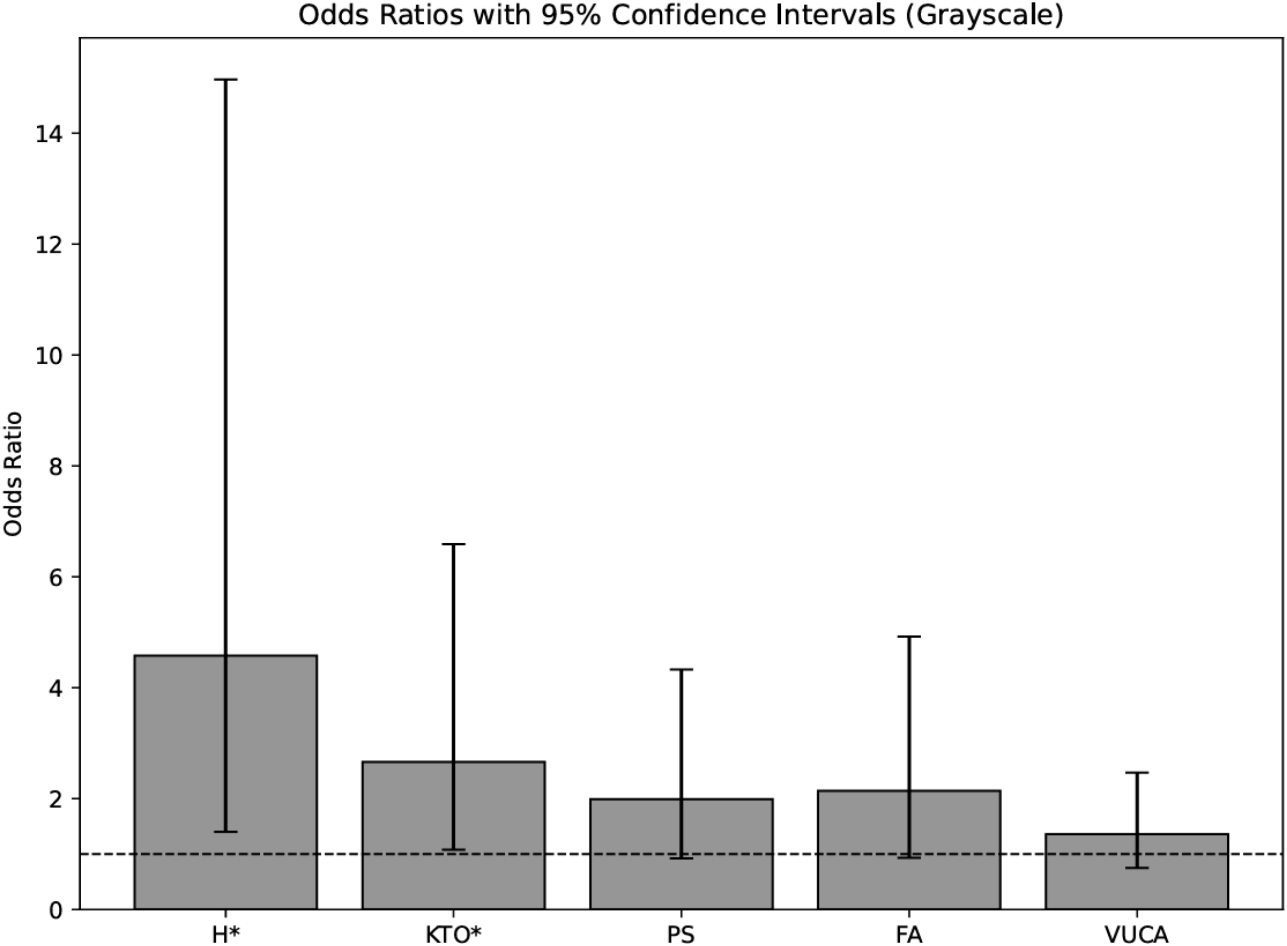
Odds ratios for psychological combat readiness for commando qualification after 24 months. PS, Psychological Stability; FA, Functional Work Attitude; KTO, Comradeship and Team Orientation; H, Hardiness; OR, Odds Ratio; 95% CI, Confidence Interval for the Odds Ratio, Significance level: p <.05 (*).

The remaining PCR predictors *PS*, *VUCA competence*, and *FA* did not reach statistical significance (p > .05). However, *PS* (*p* = .081) and *FA* (*p*= .074) showed directional tendencies suggesting potential relevance in larger samples or extended models. All predictor coefficients and confidence intervals are summarized in **Table 4**, with **Figure 6** illustrating the respective odds-ratio distributions.

**Table 4:** PCR predictor of commando qualification after 24 months.

Predictor	β coefficient	P-values	Odds-ratio	95 %-CI (OR)
H	+1.52	.012*	4.58	[1.40, 14.97]
CTO	+0.98	.034*	2.66	[1.08, 6.59]
PS	+0.69	.081	1.99	[0.92, 4.33]
FA	+0.76	.074	2.14	[0.93, 4.92]
VUCA	+0.31	.305	1.36	[0.75, 2.47]

PS, Psychological Stability; FA, Functional Work Attitude; CTO, Comradeship and Team Orientation; H, Hardiness; OR, Odds Ratio; 95% CI, Confidence Interval for the Odds Ratio, Significance level: p <.05 (*).

To evaluate long-term changes over the final training year (15 vs. 24 months), group differences in PCR factors were examined at the end of the 24-month training cycle. No significant differences were found in *PS*, *VUCA competence* or *CTO*. However, the experimental group showed unadjusted advantages in Hardiness (F = 4.08, *p* < .05, η^2^_p_ = .08) and Functional Work Attitude (F = 5.26, *p* < .05, η^2^_p_ = .09). As in Study 1, Benjamini–Hochberg FDR corrections were applied to all mean comparisons, and none of the unadjusted effects remained statistically significant after FDR adjustment. The descriptive differences nonetheless indicate stable tendencies consistent with the overall pattern of Study 1. Mean values for the 24-month assessment were 4.43 (*SD*= 0.19) for Hardiness and 4.42 (*SD* = 0.24) for FA in the experimental group, compared to 4.26 (*SD* = 0.31) and 4.21 (*SD* = 0.35) in the control group.

Finally, baseline PCR differences between candidates who later qualified and those who did not were examined to assess potential selection biases. None of the PCR dimensions showed statistically significant baseline differences (*Hardiness*: t = 0.867, *p*= .40; *PS*: t = 0.610, *p*= .55; *VUCA*: t = 0.462, *p* = .650; *FA*: t = 1.095, *p*= .291; *CTO*: t = 0.247, *p*= .807), indicating that qualification outcomes were not predetermined by initial psychological PCR-profiles in this sample.

## 4 Discussion

### 4.1 Interpretation

The present research aimed to empirically validate psychological components of Psychological Combat Readiness (PCR) in the context of the basic SOF training and qualification of the German Special Forces Command (KSK) and critically evaluate its applicability. While study 1 explored whether targeted PCR training during basic SOF military education could enhance both psychological readiness and operational performance, study 2 examined the predictive value of specific psychological traits for commando success.

Across Study 1 and Study 2, the findings consistently demonstrate that PCR is a meaningful predictor of success in high-stakes military qualification and training environments. In particular, *Hardiness, Functional Work Attitude (FA)* and *Comradeship and Team Orientation (CTO*) emerged as statistically significant factors influencing both the likelihood of passing the Commando Qualification and the development of performance during basic SOF training. Soldiers who scored higher in these domains showed a markedly increased probability of successfully completing the basic training which is in accordance with and replicates the results of extant studies (60, 75, 76).

Moreover, the results indicate that PCR is not a static trait but can be enhanced through targeted psychological training. Participants in the experimental group who received structured PCR training demonstrated improvements in key psychological dimensions such as *Hardiness, FA*, and *Performance Motivation*. These psychological gains were accompanied by superior performance in military-tactical domains, including Close Quarters Battle (CQB), advanced CQB and tactical hand-to-hand combat training, suggesting that psychological interventions can translate into operational effectiveness like in previous studies (74, 77). Moreover, *Hardiness* and *FA* were found to be significant predictors of performance outcomes in advanced CQB for the experimental group, highlighting the influence of psychological intervention through PCR training (1).

Study 2 further revealed the long-term relevance of *Hardiness* and *CTO* for determining final suitability as a Special Forces Operator. The candidates of the experimental and control group with high scores in these areas were significantly more likely to be classified as “suitable,” with strong odds ratios (Hardiness: OR = 4.58; CTO: OR = 2.66). These findings align with prior research emphasizing the role of psychological *Hardiness* and social cohesion in elite military contexts (8, 28, 75).

Interestingly, constructs such as *Psychological Stability*, *VUCA Competence*, and *FA* did not show significant predictive value in the qualification stage. This may be attributed to contextual factors, limited variance within the sample, or statistical suppression effects. *FA*, *PS* and *VUCA Competence* failed to reach statistical significance, contradicting some prior findings (9) and theoretical models (78).

The increase in *CTO* observed in the control group may reflect compensatory group dynamics under conditions of lower individual performance (1, 8, 13). Additionally, differences in group composition and prior experience may have influenced outcomes. It is important to note that both groups received required distinct psychological training modules during the basic SOF training, which could have introduced confounding effects. These modules were based on sport psychology frameworks (20, 64) and aimed to enhance performance-related psychological traits.

Taken together, the results from Study 1 and Study 2 provide empirical support for the predictive validity of specific psychological characteristics and underscore the practical relevance of psychological interventions in the training and selection of special operations personnel. The findings support existing theories on the importance of motivational and interpersonal competencies in high-stress environments (75, 79–83).

Furthermore, the studies highlight that PCR can be stabilized and even enhanced through structured interventions during basic SOF training. This is particularly noteworthy given the typical decline in self-assessed psychological resources under the demanding conditions of a 24-month basic SOF training program (1, 80, 84). The ability to maintain or improve psychological readiness under such conditions suggests that PCR training may be a promising approach for sustaining performance in elite military units.

### 4.2 Current research overlaps

The results of the current study into Psychological Combat Readiness (PCR) successfully replicate and extend extant research findings. Study 1 demonstrated that psychological training interventions exert a positive influence on soldiers’ performance capabilities (31, 37, 85, 86). Specifically, our study demonstrates that training psychological competences significantly improved shooting performance in Close Quarters Battle (CQB), aligning with results from Williams et al. (51)Ytterbøl et al. (74) and da Silva et al. (26). While Ytterbøl et al. (74) documented enhanced marksmanship in a Norwegian SOF sniper course, Williams et al. (51) reported improvements in basic marksmanship following psychological interventions and da Silva et al. (26) showed resilience predicts shooting performance in the enhanced marksmanship course of the basic SOF training of the KSK.

Study 1 also confirmed the positive effects of *Hardiness* on close combat training during basic military education among Dutch recruits. Lo Bue et al. (32) found that after 22 weeks of basic training, soldiers with higher *Hardiness* scores remained in service and showed strong correlations between initial *Hardiness* levels and performance in military hand-to-hand combat. However, their study relied solely on instructor ratings of competence, without assessing physical performance metrics. In contrast, Study 1 incorporated objective physical indicators such as punch and kick force, speed, accuracy, and instructor evaluations, offering a more comprehensive assessment of combat readiness (e.g. 1).

Furthermore, Study 1 demonstrated that specialized psychological performance programs can be effectively implemented within special forces training, provided sufficient time and personnel resources—such as the involvement of a dedicated performance psychologist—are available. The study also supported Maddi et al.’s (36) assertion that *Hardiness* is trainable. The experimental group showed significant improvements, compensating for initial disparities during basic training within 15 months and surpassing baseline levels after 24 months (37).

Additional research, such as the longitudinal study by Hystad et al. (87) involving Norwegian officer candidates over three years, found no significant changes in *Hardiness* scores. However, participants in that study did not receive targeted *Hardiness* training aimed at enhancing this trait.

In Study 1, *Functional Work Attitude* emerged as a robust predictor for military performance: upcoming commando soldiers exhibiting goal-oriented, structured, and performance-driven behavior had a significantly higher probability of passing the advanced CQB course and showed higher results in the hand-to-hand combat training. These findings are consistent with performance-relevant personality traits (88–90). In military contexts, *FA* is considered a key success factor, supporting both short-term performance and long-term operational readiness (1, 3).

Study 2 further confirmed that *Hardiness* plays a decisive role in long-term aptitude assessments, such as the two-year basic SOF training program of the German Army Special Forces Command (KSK). Candidates with higher levels of *Hardiness* were better equipped to cope with the demanding nature of the training and were more likely to complete it successfully. These findings are consistent with those of Bartone et al. (75) and Lo Bue (91), both of whom emphasized the relevance of *Hardiness* in special forces selection processes.

Bartone et al. (75) investigated whether *Hardiness* could predict successful completion of a challenging selection course for the U.S. Army Special Forces. Using the Dispositional Resilience Scale (DRS-15), they assessed 1,138 male candidates and found that those who completed the course had significantly higher *Hardiness* scores than those who did not. Logistic regression analysis revealed that each additional point on the Hardiness scale increased the likelihood of successful course completion by approximately 3.3%. These results underscore *Hardiness* as a critical psychological resource for success in extreme performance environments—findings that mirror those of Study 2.

Importantly, *Hardiness* is measurable not only during training but also prior to its commencement, suggesting its potential utility as a selection criterion due to its relative stability as a personality trait.

In addition to *Hardiness*, *Comradeship and Team Orientation (CTO)* was found to significantly influence final suitability for special forces roles (8, 13, 51, 81, 92). *CTO*, defined as the ability to collaborate and demonstrate camaraderie within a team, plays a crucial role in how candidates adapt to dynamic interpersonal challenges—especially under stress or when making complex decisions in team settings (47). Beyond individual performance orientation, the social dimension therefore also proved critical: These findings highlight the importance of social cohesion in military units, as described by Siebold (8) and MacCoun et al. (47). Social integration, trust, and cooperative teamwork are recognized as essential protective and performance-enhancing factors in high-stress environments.

Taken together with findings from Study 1, it can be concluded that *Hardiness* is a psychological construct that can be assessed before, during, and after training, serving as a reliable predictor of successful completion of basic SOF training (75). The interplay between *Hardiness* and *CTO* highlights the importance of holistic selection procedures that consider both psychological resilience and cognitive abilities (26, 33) to identify and prepare suitable candidates for special operations roles.

Overall, the findings showcase the importance of targeted psychological training interventions for enhancing mission-critical competencies in special operations roles. *Hardiness* and *CTO* emerged as significant predictors of long-term suitability for special forces, while the combination of *FA* and *Hardiness* proved to be a particularly powerful predictor of military performance (advanced CQB) during basic SOF training.

### 4.3 Limitations

The two studies conducted on Psychological Combat Readiness (PCR) among candidates for the German Army Special Forces Command (KSK) provide robust insights into psychological predictors, training effects, and performance-related associations within the context of elite military operations. Notably, there are currently no other publicly available scientific investigations focused specifically on German SOF (3, 26). A major strength of this research lies in the development and evaluation of tailored psychological training programs for special operations personnel (1, 73, 74, 93). Furthermore, this work represents the first empirical study to introduce a conceptual psychological model centered on PCR (3). Previous research in this domain has relied primarily on general resilience frameworks (15, 20, 64). As such, the present study addresses a critical gap in literature and underscores the relevance of Psychological Combat Readiness in military selection and training.

However, several methodological limitations must be considered when interpreting the findings. A key constraint is the relatively small sample size, which reflects the high selectivity of the selection and the demanding nature of the basic SOF training program. This limitation is common in special forces research (33), and only large-scale U.S. army and marine studies such as Bartone et al. (75) and Farina et al. (60) have been able to overcome it due to broader personnel resources. Additionally, in Study 1, many participants were unavailable for follow-up due to assignments in various domestic and international training programs, which significantly reduced statistical power and limited the generalizability of the results (e.g. 75).

The studies employed quasi-experimental longitudinal designs (94). While these approaches allow for initial predictive insights, they do not permit strong causal inferences (95). Accordingly, the results should be interpreted as correlational (96). Both studies had to accommodate existing military training structures to avoid disrupting operational schedules—a constraint that will likely persist due to the prioritization of training and deployment within the Special Forces Command.

Another limitation concerns the control group in Study 1, which also received psychological training components, including relaxation techniques and sport psychology modules (63, 64). This overlap makes it difficult to isolate the effects of the PCR-specific interventions. Future studies would benefit from a clearer separation of training content to enhance internal validity.

Group composition also presents a potential confounding variable. The study groups differed in certain PCR components prior to the interventions, complicating the interpretation of training effects. Moreover, group dynamics—such as platoon cohesion or leadership influences—may have impacted outcomes (8, 82).

We did not systematically assess prior military experience, training history, or other relevant background factors that may have influenced psychological readiness or performance outcomes. Such pre-existing differences could have contributed to observed effects and should be considered in future research.

External validity is similarly limited. The sample consisted exclusively of male soldiers from a highly selective military unit (48, 96). Nevertheless, the results may serve as comparative data for other German and international Special Operations Forces. Additionally, the group- and individual-level assessment of PCR components within the German Army Special Forces Command offer a valuable tool for evaluating psychological performance capacity.

Self-selection of participants represents another relevant factor. Only highly motivated and pre-screened individuals voluntarily participate in the SOF selection, which may introduce social-psychological biases into the study outcomes (74, 97). Despite this, Farina et al. (80) documented significant psychological changes during special forces training, including declines in resilience over a two-year period. To avoid relying solely on self-reported data, Michels and Mair are currently developing an external PCR assessment instrument to be used by instructors during basic SOF training. The integration of such data beyond self-reports represents a meaningful methodological enhancement (1, 2).

Study 1 may also be subject to expectancy effects. Participants’ anticipatory beliefs about the study’s purpose or hypotheses (98), as well as interactive influences from experimenters and the performance psychologist (99), could have introduced systematic bias. Such effects may lead to behavioral changes that are not solely attributable to the intended experimental manipulation. For example, the training modules and the expectations conveyed by the psychologist may have shaped participant responses. It remains unclear whether the induced experimental conditions aligned with participants’ subjective expectations. Discrepancies between intended and perceived manipulation could affect internal validity. Additionally, communication between the experimental and control groups regarding intervention methods may have led to shared expectancy effects. Future research should incorporate stronger methodological controls, such as manipulation checks, to assess individual expectation configurations. Another consideration is that different psychologists administered the training modules in Study 1, which may have introduced variability.

In the analyses of Studies 1 and 2, logistic regression models were applied without controlling for relevant variables such as physical fitness, educational background, prior military experience or injuries sustained during training. This omission may have impacted both internal and external validity. Future investigations should consider randomized experimental designs, larger and more diverse samples, and standardized multi-perspective measurement tools to enhance the explanatory power of findings related to Psychological Combat Readiness in special forces and infantry units.

### 4.4 Practical implications of psychological combat readiness

The two studies conducted on Psychological Combat Readiness (PCR) among candidates for the German Army Special Forces Command provide practice-relevant insights for qualification, training, and performance optimization and enhancement in elite military contexts. The findings demonstrate that psychological training interventions—particularly those aimed at strengthening *Hardiness*, *Functional Work Attitude (FA)*, and *Comradeship and Team Orientation (CTO)—*can exert a significant influence on soldiers’ operational capabilities and suitability (2, 28, 37, 85).

Taken together, these findings suggest that psychological components of the PCR concept such as *Hardiness*, *Functional Work Attitude*, and *CTO* are not only trainable but also predictive of training success (100). They should therefore be systematically integrated into selection and training processes. The studies further demonstrate that psychological training interventions are feasible within the structural constraints of military training institutions—provided that appropriate resources are allocated. For example, Ytterbøl et al. (93) describe the deployment of a performance psychologist with Norwegian special forces, where mental performance optimization programs were conducted in operational environments.

Since 2018, the German Army Special Forces Command has operated its own Human Performance Optimization (HPO) program, and since 2020, a dedicated Human Performance Systems Cell has supported tactical performance optimization through interdisciplinary measures—spanning sports science, psychology, medicine, and chaplaincy (101). As of 2023, a designated HPO psychologist oversees the psychological component, delivering 15 structured modules to strengthen Psychological Combat Readiness. Additionally, active operators undergo psychological assessments—including PCR screening—every 18 months, receiving individualized feedback to enhance mental performance (101). This marks the formal integration of PCR as both a screening tool and a training measure within the KSK since 2023, in line with recommendations by Ytterbøl et al. (20, 93 and Lo Bue (91). The guiding principle for special operations forces must be: *select them well—and train them even better*. From a psychological perspective, the two-year basic SOF training phase must be strategically utilized to ensure long-term operational success by optimally preparing future commando operators for the demands of their specialized roles. Therefore, the Psychological Combat Readiness concept and training is part of the German SOF basic training in the German Army Special Forces Command.

In sum, the evidence supports the conclusion that PCR is a central success factor in elite military roles. Its systematic assessment, enhancement, and integration into existing structures can contribute meaningfully to sustaining and improving the performance and operational readiness of special forces personnel—select them well, train them better!

### 4.5 Strategic integration of psychological interventions in SOF basic training

The guiding principle for special operations forces must be: *select them well—and train them better*. From a psychological standpoint, the two-year basic training phase in SOF must be strategically leveraged to ensure long-term operational success and mental performance of the operator. This period is not merely a physical conditioning program—it is a critical developmental window for cultivating the psychological competencies that underpin elite performance in high-risk environments.

Modern SOF roles demand more than tactical proficiency; they require mental resilience and robustness, structured motivation, and interpersonal cohesion—competencies that enable soldiers to remain effective under extreme stress, adapt to volatile and violent conditions, and function seamlessly within tightly knit teams. As Bartone et al. (85) emphasized, “psychological hardiness is a key factor in soldier performance under pressure,” and its development should be a deliberate component of training. Similarly, Maddi et al. (28) demonstrated that hardiness traits such as commitment, control, and challenge can be enhanced through structured interventions, leading to improved performance and retention in demanding military contexts.

The German Army Special Forces Command (KSK) has responded to these demands by integrating the Psychological Combat Readiness concept into its basic SOF training curriculum. This includes modular psychological training delivered by Human Performance Optimization (HPO) psychologists, covering dimensions such as *Hardiness*, goal-directed behavior, and social competence. As Ytterbøl et al. (93) noted in their work with Norwegian SOF units, “embedding psychological training within operational environments enhances both individual readiness and collective mission success.”

In sum, the psychological development of future commando operators must be treated with the same strategic priority as physical and tactical preparation. By embedding Psychological Combat Readiness into the core of SOF training, military organizations can foster resilient, motivated, and cohesive operators—capable of thriving in the most complex and demanding operational scenarios.

## 5 Conclusion

The present study demonstrates that integrating psychological training modules into special forces education is not only feasible but also performance-relevant. Psychological Combat Readiness functions as a *key resource* that supports both individual mission readiness and team-based effectiveness in complex, dynamic operational scenarios. These findings provide not only empirical evidence for the importance of psychological factors in military contexts but also concrete recommendations for the continued development of selection and training procedures within special operations forces.

## Data Availability

The datasets are not available to the public, to secure the operational procedures of the German Army Special Forces Command. The dataset is classified. Queries should be directed to the corresponding authors.
